# TNF-α Regulates the Effects of Irradiation in the Mouse Bone Marrow Microenvironment

**DOI:** 10.1371/journal.pone.0008980

**Published:** 2010-02-01

**Authors:** Ana Sofia Cachaço, Tânia Carvalho, Ana Cristina Santos, Cátia Igreja, Rita Fragoso, Catarina Osório, Manuela Ferreira, Jacinta Serpa, Sofia Correia, Perpétua Pinto-do-Ó, Sérgio Dias

**Affiliations:** 1 Angiogenesis Laboratory, Centro de Investigação em Patobiologia Molecular (CIPM), Instituto Portugues de Oncologia de Francisco Gentil, Lisboa, Portugal; 2 Departamento de Anatomia Patologica, Faculdade de Medicina da Universidade de Lisboa, Lisboa, Portugal; 3 Instituto Gulbenkian de Ciencia, Oeiras, Portugal; 4 Divisão de Biomateriais, Instituto de Engenharia Biomédica (INEB), Universidade do Porto, Porto, Portugal; University of Barcelona, Spain

## Abstract

**Background:**

Secondary bone marrow (BM) myelodysplastic syndromes (MDS) are increasingly common, as a result of radio or chemotherapy administered to a majority of cancer patients. Patients with secondary MDS have increased BM cell apoptosis, which results in BM dysfunction (cytopenias), and an increased risk of developing fatal acute leukemias. In the present study we asked whether TNF-α, known to regulate cell apoptosis, could modulate the onset of secondary MDS.

**Principal Findings:**

We show that TNF-α is induced by irradiation and regulates BM cells apoptosis *in vitro* and *in vivo*. In contrast to irradiated wild type (WT) mice, TNF-α deficient (TNF-α KO) mice or WT mice treated with a TNF-α-neutralizing antibody were partially protected from the apoptotic effects of irradiation. Next we established a 3-cycle irradiation protocol, in which mice were sub-lethally irradiated once monthly over a 3 month period. In this model, irradiated WT mice presented loss of microsatellite markers on BM cells, low white blood cell (WBC) counts, reduced megakaryocyte (MK) and platelet levels (thrombocytopenia) and macrocytic anemia, phenoypes that suggest the irradiation protocol resulted in BM dysfunction with clinical features of MDS. In contrast, TNF-α KO mice were protected from the irradiation effects: BM cell apoptosis following irradiation was significantly reduced, concomitant with sustained BM MK numbers and absence of other cytopenias. Moreover, irradiated WT mice with long term (≥5 months) BM dysfunction had increased BM angiogenesis, MMPs and VEGF and NFkB p65, suggestive of disease progression.

**Conclusion:**

Taken together, our data shows that TNF-α induction following irradiation modulates BM cell apoptosis and is a crucial event in BM dysfunction, secondary MDS onset and progression.

## Introduction

Tumour Necrosis Factor-α (TNF-α) is a pro-inflammatory cytokine secreted by activated macrophages and T lymphocytes, but also by keratinocytes and fibroblasts [reviewed in 1]. Its role in inflammatory processes is based both on tissue destruction and subsequent recovery of tissue homeostasis [2]. However, its role in carcinogenesis is more controversial, since it can selectively cause apoptosis of tumour endothelial cells via a caspase cascade [reviewed in 3], but can also promote tumour growth and metastasis, probably via nuclear factor NF-κB activation [2; reviewed in 1]. The link between chronic inflammation and cancer is well demonstrated in the TNF-α-deficient mice model, which is resistant to skin carcinogenesis. Conversely, the presence of TNF-α on wild-type (WT) animals increased their susceptibility to tumour promotion [4]. TNF-α deficiency was associated with reduced MMP9 expression, which correlated with reduced keratinocyte migration, limiting skin tumour development [5]. In addition, several studies have shown a role for TNF-α-induced MMPs in tumour progression and invasiveness [6]–[8].

TNF-α has also been implicated in BM diseases such as Fanconi anemia [9], [10], aplastic anemia [11], [12] or myelodisplasic syndromes (MDS). Bone marrow failure in MDS involves apoptosis induction, which may involve TNF-α [13]; persistent BM dysplasia following benzene exposure has also been associated with TNF-α polymorphisms [14]. Nevertheless, the therapeutic efficacy of anti-TNF-α approaches, tested in patients with *de novo*, or primary, MDS, has been relatively modest [15]–[17].

Despite some evidence pointing for a putative role of TNF-α in regulating BM disease onset, studies exploiting its involvement in secondary (irradiation-induced) BM failure/MDS are lacking, and were the subject of the present study. Patients with secondary MDS (which develop following chemotherapy or radiotherapy for other cancers) have worse prognosis than primary MDS [18], and as such it represents a serious complication of cancer treatments. The data shown in this report identify TNF-α as a key cytokine in the BM microenvironment, important for cell apoptosis, sensitivity to irradiation, BM dysfunction and secondary MDS onset and progression. Anti-TNF strategies may be beneficial for the treatment of subsets of patients with BM dysfunction/secondary MDS.

## Results

### Single Irradiation Induces BM Cell Apoptosis and TNF-α Production

**Figure 1:**
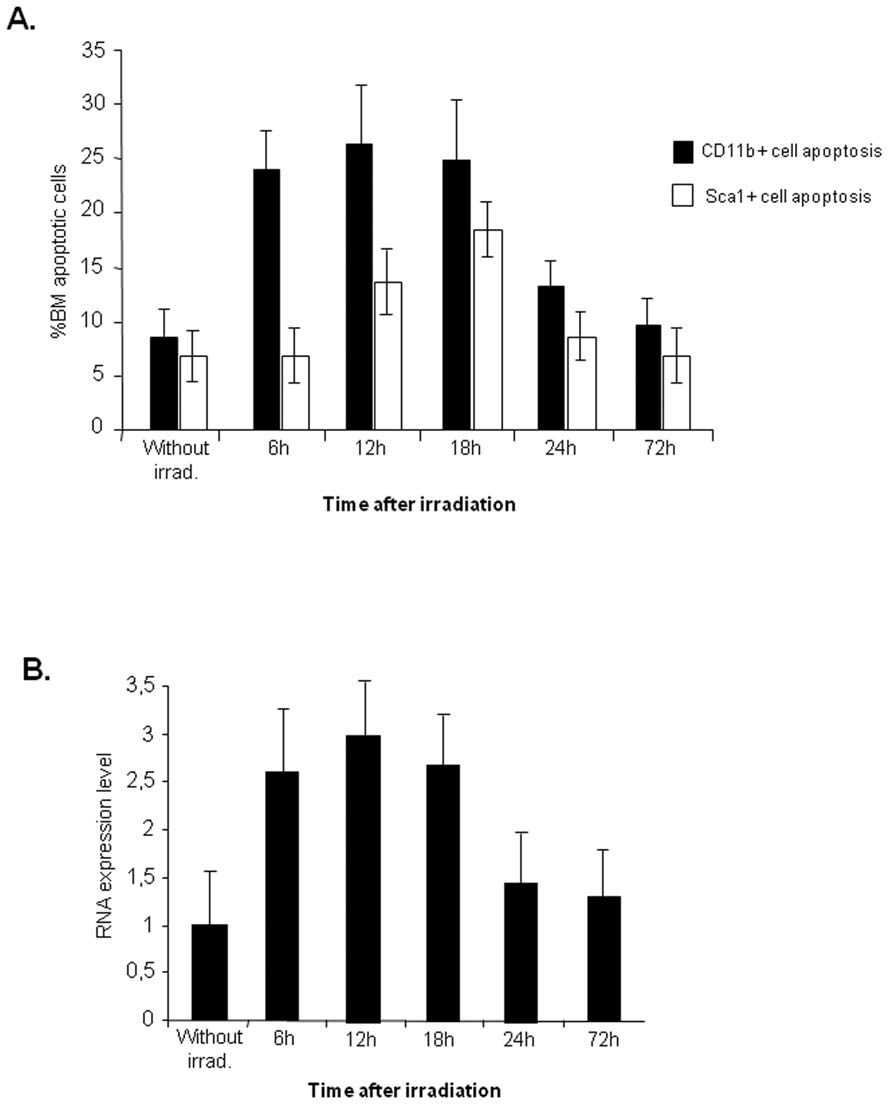
Irradiation induces BM cell apoptosis which correlates with an increase in TNFα expression. **A.** Flow cytometry analysis of WT mouse BM cells shows a rapid increase in CD11b+ and Sca1+ cells apoptosis 6-12 hours after sub-lethal irradiation, returning to normal levels after 72 hrs. **B.** TNF-α quantification by RQ-PCR on the same samples indicates a rapid increase in TNF-α mRNA after irradiation. The results shown were obtained from 8 independent experiments using 6 animals per time point.

We reasoned an increase in BM TNF-α might correlate with BM cell apoptosis induced by irradiation. To test this hypothesis, we analysed the effects of sub-lethal irradiation in BM cell turnover over a 3 day (72 h) period (“short-term irradiation effect”). As shown in Figure 1A, BM CD11b+ (myeloid) and Sca1+ (haematopoietic precursors) cell apoptosis increases in the initial 18 hours following irradiation, decreasing to control (non-irradiated) levels by 72 hours. As determined by RQ-PCR, the BM levels of TNF-α show a similar trend, increasing in the initial 18 hours and returning to control levels after 72 hours (Figure 1B).

### TNF-α Induces *In Vitro* BM Cell Apoptosis

**Figure 2:**
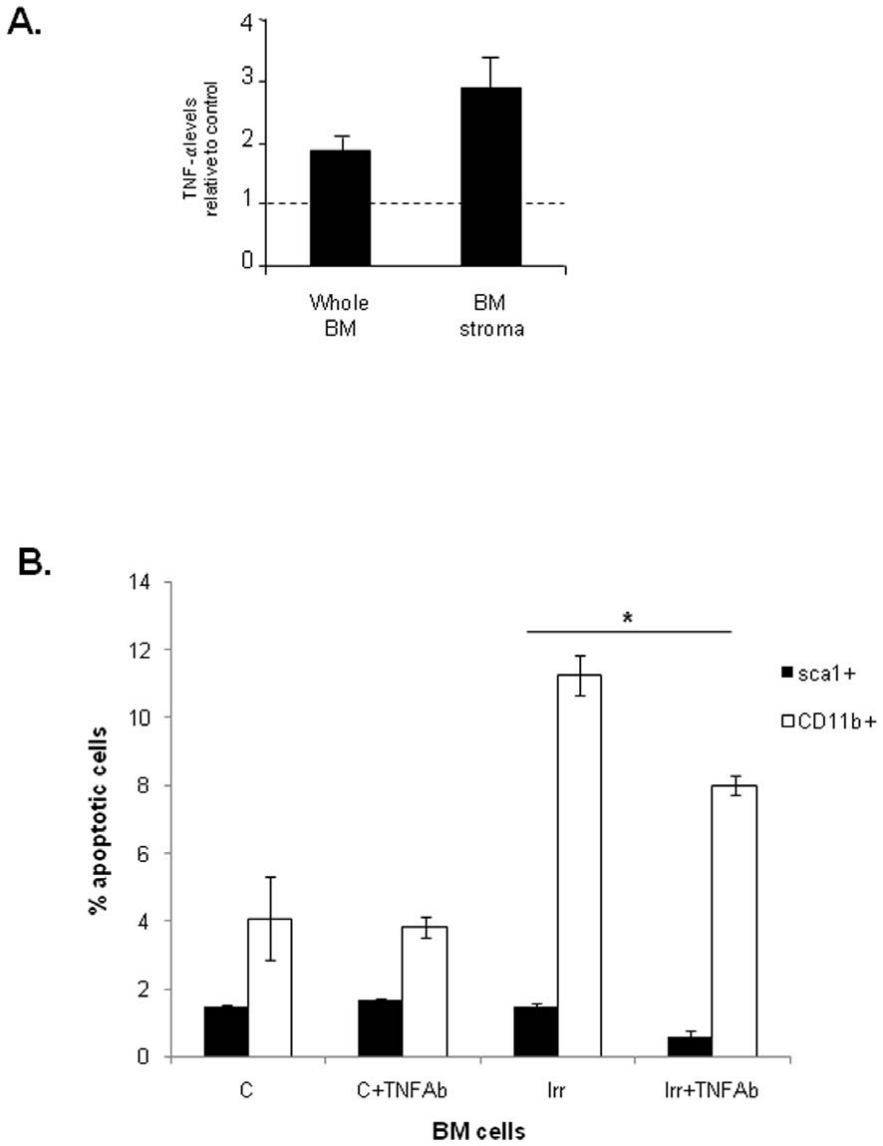
Irradiation-induced TNF-α release results in BM cell apoptosis *in vitro*. **A.** TNF-α protein measured by ELISA indicates an increase in TNF-α levels after irradiation, both in supernatants of cultured total BM mononuclear cells and stromal cells. **B.** Apoptosis of BM cells, incubated with the supernatants obtained in A, untreated or treated with an anti-TNF-α antibody. The presence of TNF-α antibody significantly decreases cell apoptosis after irradiation, both for Sca1+ and CD11b+ cells. The results shown were obtained from 2 independent experiments. *: p<0.05 for CD11b+ and for Sca1+.

Next we tried to find a causal relationship between the increase in TNF-α levels and the incidence in BM cell apoptosis following irradiation. For this purpose, we irradiated whole BM mononuclear cells and BM stromal cells *in vitro*, and measured the levels of TNF-α released into the culture supernatants by ELISA. As shown in Figure 2A, irradiation induces TNF-α production by whole BM and BM stroma.

Next, we hypothesized that TNF-α might be responsible for the incidence in BM cell apoptosis, and thus neutralizing its activity might exert a protective effect. Therefore, we exposed subsets of BM cells to the supernatants described earlier, and tested the protective effects of adding a TNF-α neutralizing antibody. As shown in Figure 2B, cells treated with supernatants obtained from irradiated BM cells show a significantly higher apoptotic index than those treated with the TNF-α neutralizing antibody and exposed to the same supernatants. (p<0.05 for Sca1+ and CD11b+ cells). These experiments suggest that TNF-α released into culture supernatants of irradiated BM cells induces BM cell apoptosis *in vitro*. Nevertheless, we cannot exclude other undisclosed factor(s) may also promote BM cell apoptosis in response to the irradiation stimulus.

### TNF-α KO Mice Are Partially Resistant to the Apoptotic Effects of Irradiation

**Figure 3:**
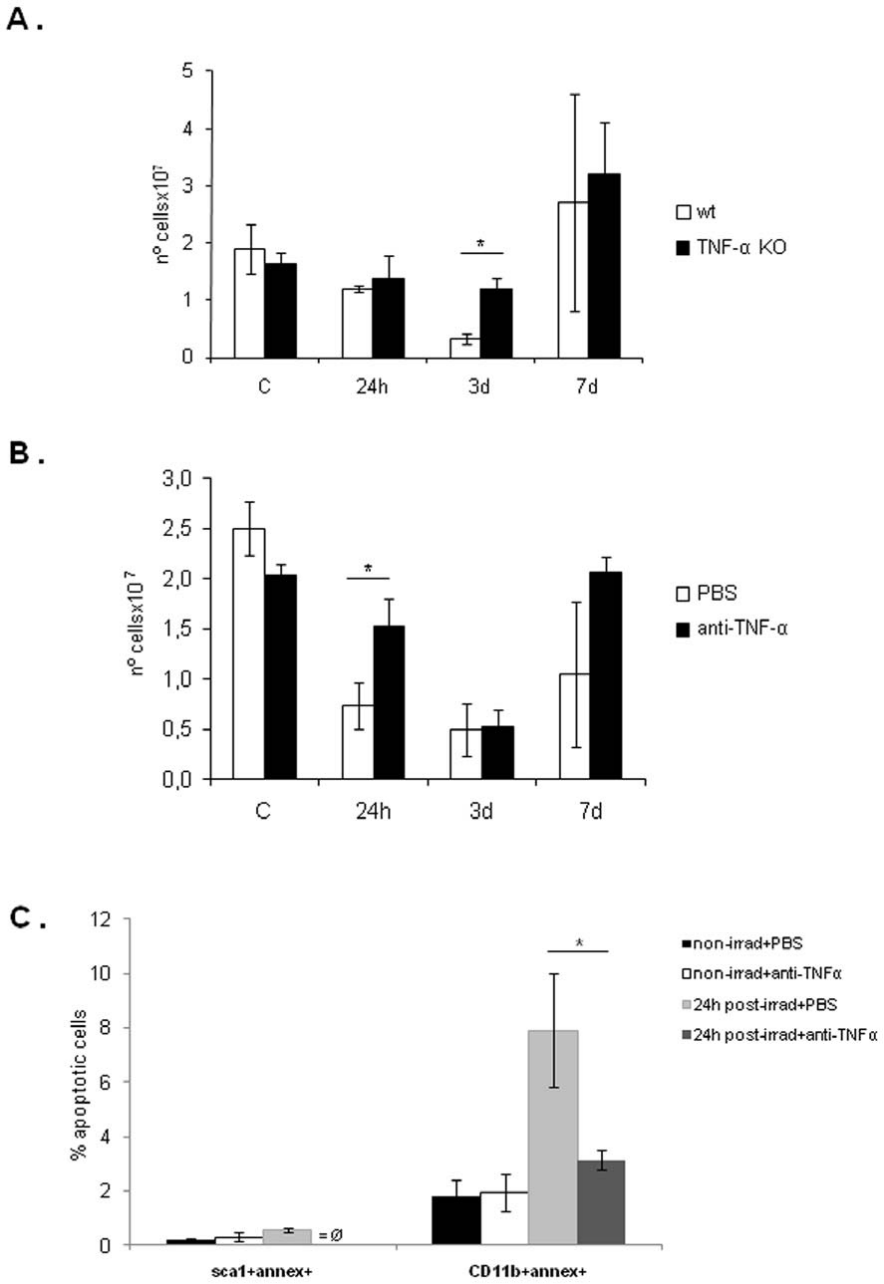
Sub-lethal irradiation reduces the number of total BM cells, an effect partially dependent on TNF-α. **A.** WT and TNF-α KO mice were sub-lethally irradiated and the total number of BM cells was counted as explained in Methods. As shown, TNF-α KO mice were more resistant to the apoptotic effects of irradiation. On day 3 following irradiation, the number of total BM cells is significantly higher in TNF-α KO mice than in WT mice. The results shown were obtained from two independent experiments, using 12 animals per experimental group and 3 animals per time point. *: p<0.05. **B.** WT mice were injected with PBS (control) or with antibody anti-TNFα prior to sub-lethal irradiation and total BM cells were counted. Like for TNF-α KO mice, anti-TNFα neutralized mice were more resistent to irradiation; here, the number of total BM cells is significantly higher (*: p<0.02) by 24 hours after irradiation than in controls. **C.** The percentage of apoptotic cells 24 hours after irradiation was obtained in control and neutralized mice by flow cytometry. Both precursor (Sca1+) and myeloid (CD11b+) cells were protected from irradiation-induced apoptosis in the anti-TNF-α treated mice, where the number of cells positive for annexin V is lower than in the controls. *: p<0.01 for CD11b+; for Sca1+ a p value could not be calculated due to the absence of Sca1+Annexin+ cells in neutralized mice. The results shown in B and C were obtained from one experiment, using 12 animals per experimental group and 3 animals per time-point.

To test the importance of TNF-α in BM apoptosis following irradiation *in vivo*, we compared the “short term irradiation effects” in wild type (WT) and TNF-α deficient (KO) mice BM content. As shown in Figure 3A, as a result of cell apoptosis, the BM cell number decreases significantly 3 days after sub-lethal irradiation, returning to control levels by 7 days. In accordance with the *in vitro* results, irradiated TNF-α KO mice were partially resistant to the apoptotic effects of irradiation (the number of BM cells is significantly higher in TNF-α KO mice BM 3 days after irradiation, p<0.05).

To overcome possible differences between the BM microenvironment of TNF-α KO and WT mice, we also tested the effects of irradiation in the BM content of WT mice treated with anti-TNF-α Ab. In Figure 3B, a marked decrease in BM cell numbers (higly significant: p<0.02) was observed in untreated (control) mice 24 hours after irradiation, comparing with Ab-treated animals. In parallel with these results, flow cytometry analysis for apoptotic cells showed that the number of Sca1/Annexin V- and CD11b/Annexin V-positive cells is significantly higher (p<0.01) in control mice in comparison with anti-TNF-α treated mice, confirming the apoptotic effect of TNF-α in both precursor and mature BM cells (Figure 3C). For the other time-points, also a minor protection from irradiation-induced apoptosis is observed in anti-TNF-α treated mice (data not shown).

Taken together, these experiments suggest irradiation induces TNF-α production in the BM, and that the released TNF-α is partially responsible for the increase in BM cell apoptosis following irradiation.

### 3-Cycle Irradiation Protocol Induces BM Dysfunction, Suggestive of MDS

**Figure 4:**
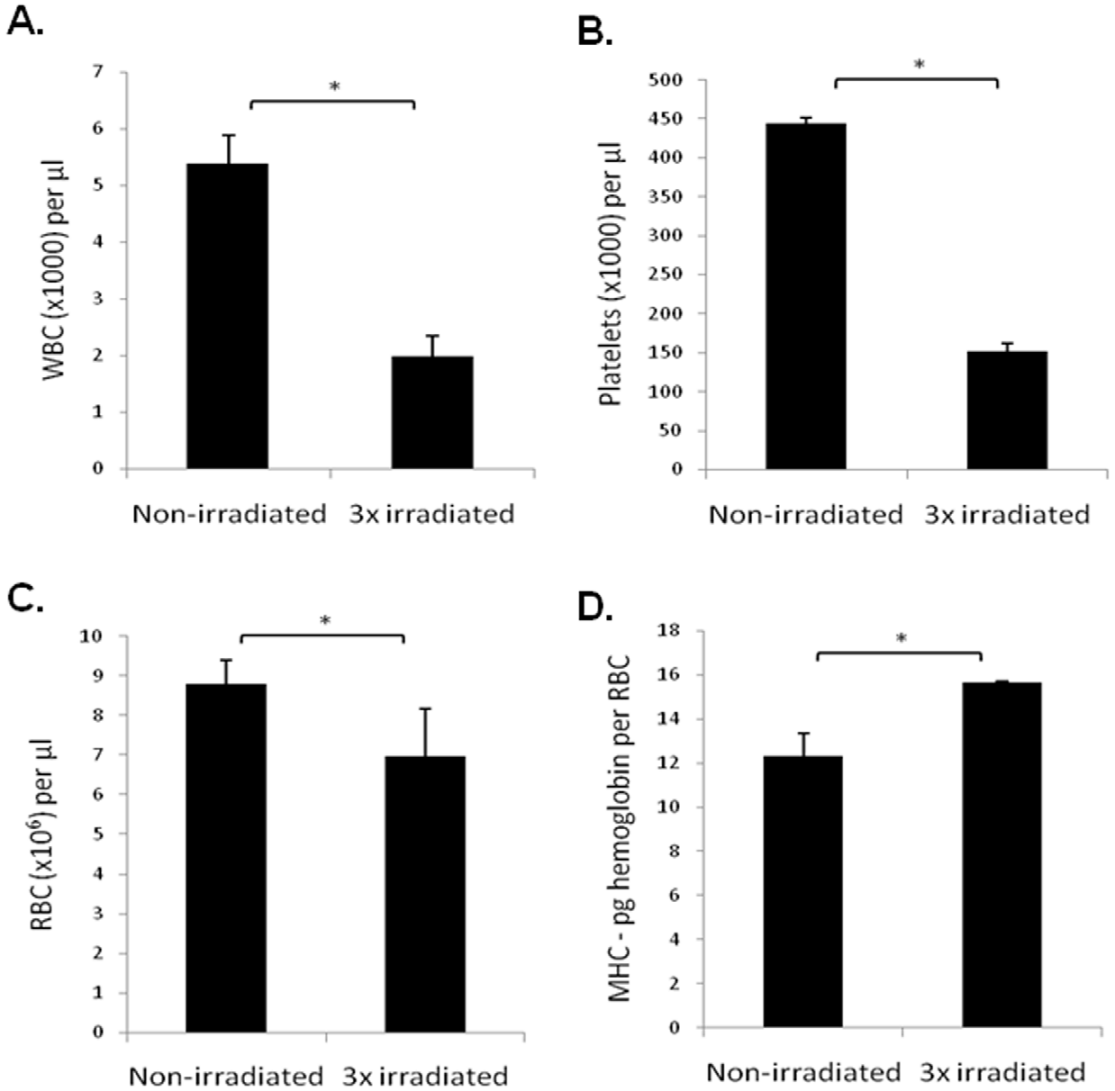
A 3-cycle irradiation protocol induces cytopenias and macrocytic anemia in WT mice. **A**. The WBC is significantly reduced in 3x irradiated mice. **B.** The number of platelets is reduced in 3x irradiated mice. **C.** The number of RBC is significantly reduced in 3x irradiated mice. *: p<0.05. **D**. 3x irradiated mice present significantly higher MCH-pg Hemoglobin per RBC than control mice. *: P<0.05.

Next, we developed a 3-cycle irradiation protocol (to test the “long term effects of irradiation”), and characterized its effects in inducing BM dysfunction. First, we showed the irradiation protocol induces loss of microsatellite markers by BM cells, suggesting it had carcinogenic/transforming capacity (Supplementary Figure S1). Concerning the haematological phenotype induced by the irradiation protocol, as shown in Fig 4, three months after the last irradiation, 3x irradiated mice showed a significant decrease in circulating white blood cells (WBC), platelets and red blood cells (RBC). In addition, 3x irradiated mice showed a significant increase in MCH-pg Hemoglobin per RBC (Figure 4D), a phenotype usually seen in macrocytic anemia. Taken together, the concomitant clinical presentation of cytopenias, thrombocytopenia and anemia, and the incidence of cytogenetic abnormalities in 3x irradiated mice, strongly suggests this may be considered a model of BM dysfunction with clinical features of secondary MDS.

### TNF-α KO Mice Are Resistant to the Irradiation-Induced BM Dysfunction

**Figure 5:**
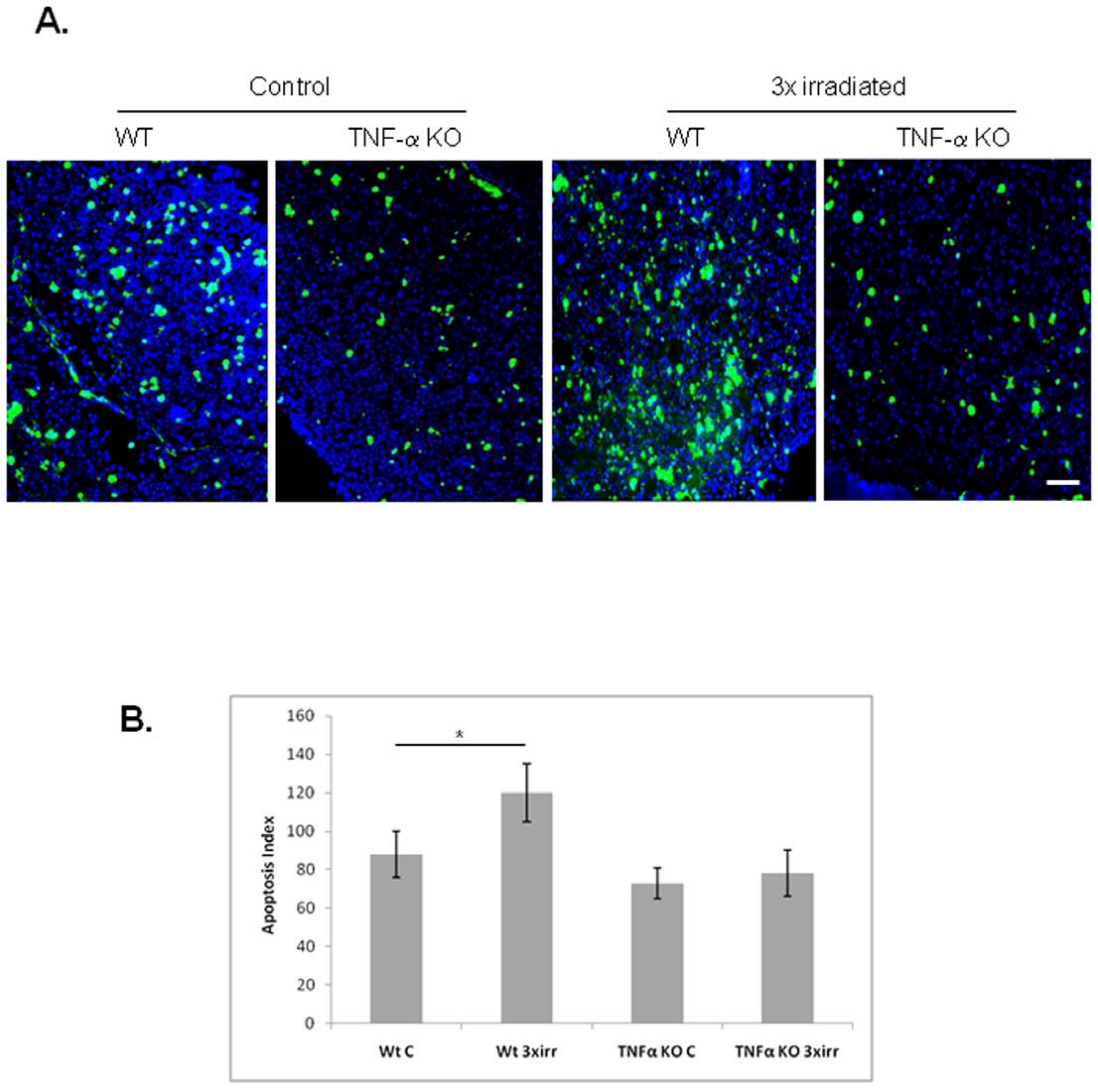
TNF-α KO mice are resistant to irradiation-induced BM cell apoptosis. **A.** TUNEL assay on BM cryosections shows increased apoptosis in 3x irradiated WT mice compared to control and to 3x irradiated TNF-α KO mice. Scale bar  = 50 µm. **B.** BM apoptotic index shows increased apoptosis in 3x irradiated WT mice. These results were obtained from 2 independent experiments, using 3 animals per experimental group. *: p<0.05.

**Figure 6:**
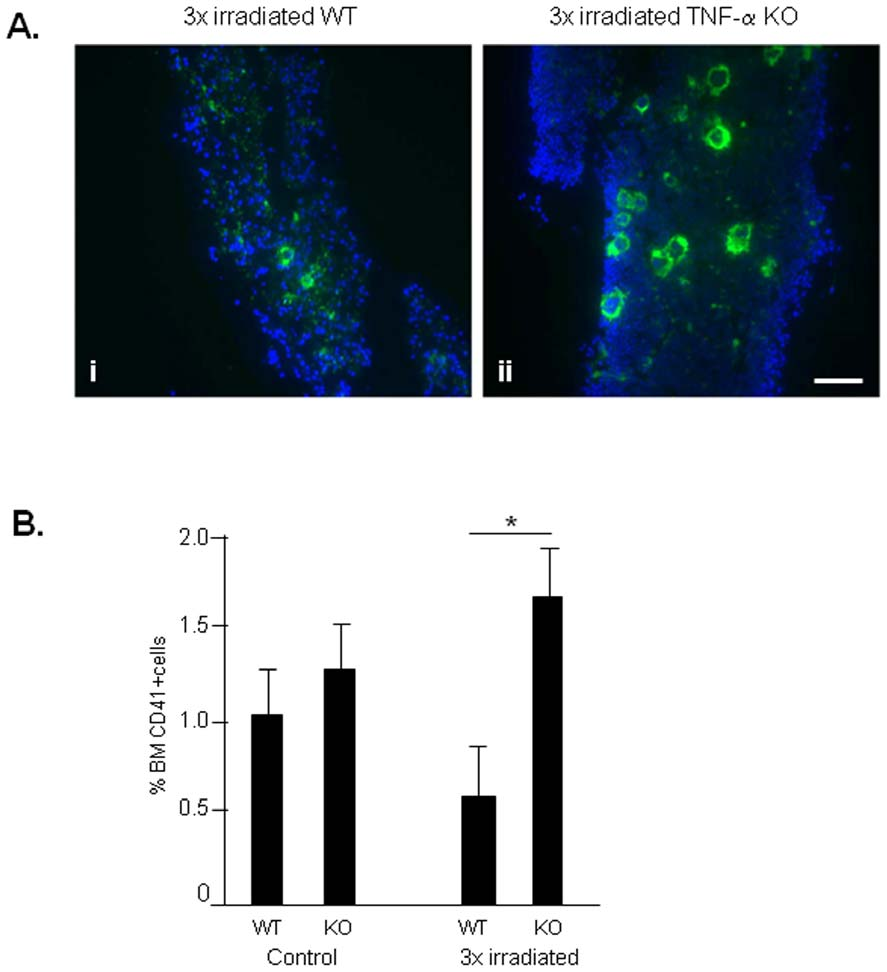
Irradiation reduces the BM MK content in WT but not TNF-α KO mice. **A.** BM cryosections of WT and TNF-α KO mice immunostained for CD-41, a megakaryocyte marker, shows increased megakaryocytes in TNF-α KO BM. Nuclei are stained in blue with Dapi. Scale bar  = 50 µM. **B.** Flow cytometry for CD41 in BM samples corroborates the idea that in the absence of TNF-α the proportion of MK is maintained or even shows a slight increase following irradiation. These results were obtained from 3 independent experiments, using 3 animals per experimental group. *: p<0.05.

Having shown TNF-α was involved in the regulation of BM apoptosis following short-term irradiation, next we tested the importance of TNF-α in our irradiation-induced model of BM dysfunction/secondary MDS. As shown in Figure 5A and B, BM cell apoptosis increased in 3x irradiated WT mice, but was significantly reduced in 3x irradiated TNF-α KO (p<0.05). Similarly, the number of MK in BM of 3x irradiated WT mice decreased significantly (correlating with the reduction in platelet levels, Figure 4), but was sustained in 3x irradiated TNF-α KO mice (Fig. 6A, B; the difference in the number of BM MK is significant, p<0.05). Circulating WBC and RBC were also higher in 3x irradiated TNF-α KO compared to 3x irradiated WT mice (data not shown). Taken together, these data suggest that TNF-α KO mice are resistant to the effects of long-term irradiation. While irradiated WT mice develop BM dysfunction with clinical features of MDS, irradiated TNF-α KO mice have sustained BM cell numbers, including MK and unchanged haematological parameters.

### WT Mice with Prolonged BM Dysfunction/MDS Present Increased BM Angiogenesis, MMPs and NFkB

**Figure 7:**
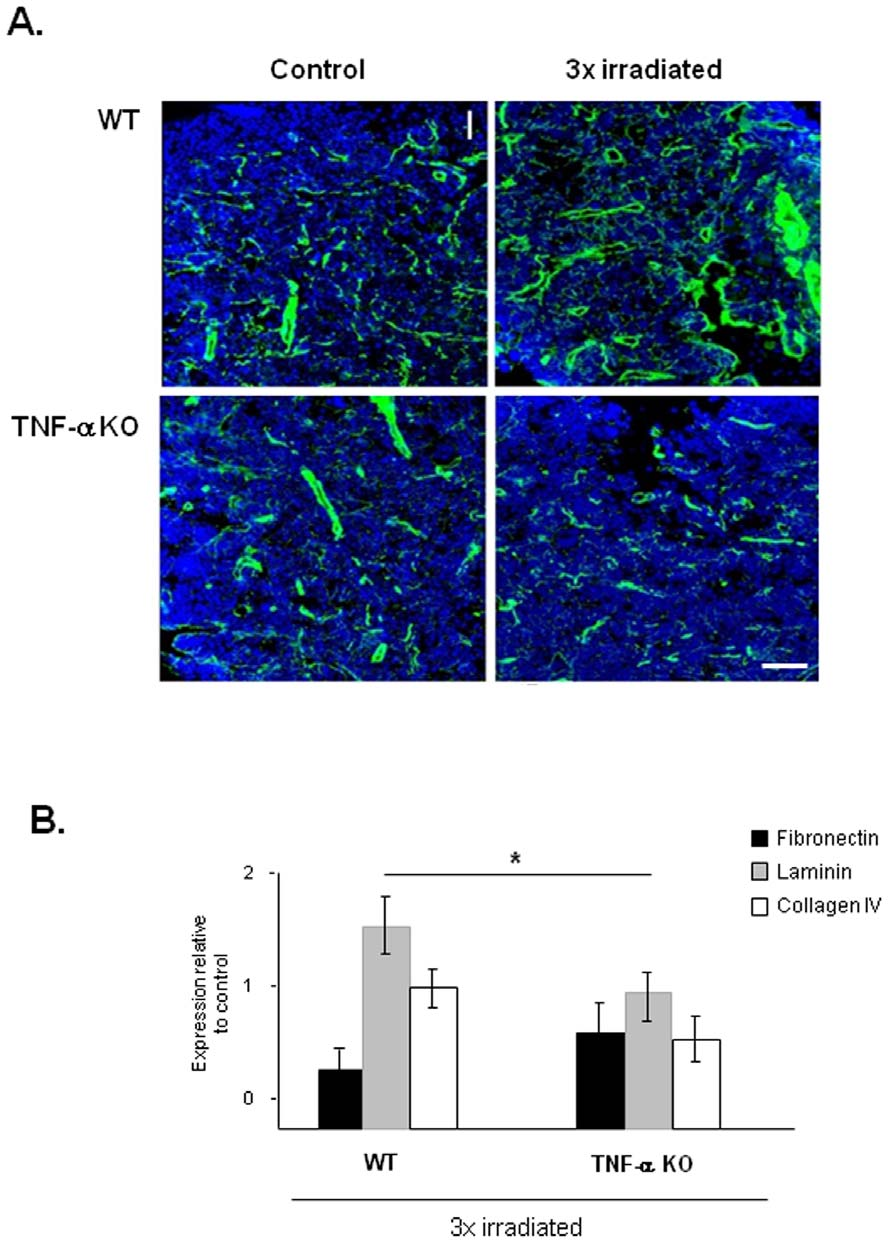
Irradiation induces BM ECM turnover in WT but not TNF-α KO mice. **A.** BM criosections of WT and KO mice immunostained for laminin. Laminin staining (green; nuclei stained in blue) which marks basement membranes shows an increase in microvessel density (more and more dilated vessels) in 3x irradiated WT mice BM (**B**) compared to control (non-irradiated) WT mice and TNF-α KO. Scale bar  = 50 µm. **B.** The expression of other ECM proteins also differs between WT mice BM and TNF-α KO mice BM. As determined by RQ-PCR, the expression of laminin and collagen IV is significantly reduced in TNF-α KO mice BM. These results were obtained from 3 independent experiments. *: p<0.05 for laminin and collagen IV.

**Figure 8:**
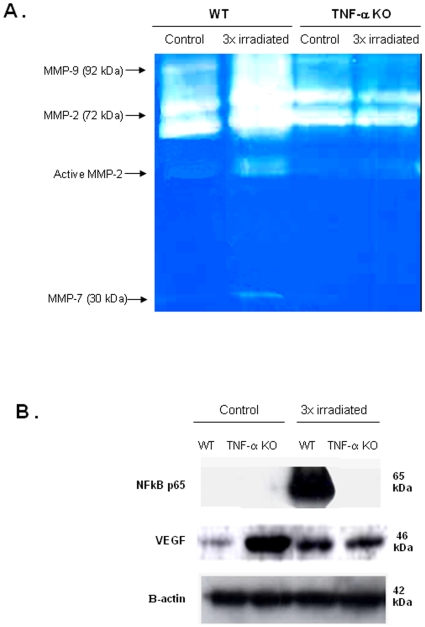
3xirradiated WT mice show MMP activation and increased NFkB and VEGF BM levels in BM extracts. **A.** Whole BM extracts obtained from control or irradiated WT and TNF-α KO mice were analysed by zymography. As shown by the classical MMP pattern seen in zymograms, MMP-9, MMP-2 and MMP-7 could be detected in BM extracts from all the mice. However, in irradiated WT mice (with prolonged MDS) the levels of MMP-9, MMP-2 and active MMP-2 and MMP-7 increased significantly. These results are representative of 3 mice per experimental group. **B.** Whole BM extracts were obtained from control or irradiated WT and TNF-α KO mice and analysed by western blotting. The results show a dramatic increase in NFkB p65 and in VEGF levels in BM extracts from irradiated WT mice with prolonged MDS, while in BM extracts from TNF-α deficient mice these parameters remain unchanged. These results are representative of 3 mice per experimental group.

MDS patients are at a higher risk of developing secondary acute leukemias [19]. In our model of irradiation-induced BM dysfunction/secondary MDS, a proportion (40%) of 3x irradiated WT mice succumb approximately 6-8 months after the last irradiation (data not shown). We characterized the BM of mice that presented an MDS-like phenotype beyond 5 months after the last irradiation, and compared it to control (non-irradiated) WT and TNF-α KO mice BM. As shown in Figure 7A, 3x irradiated WT mice had increased BM microvessel density (more and dilated vessels), indicative of increased angiogenesis. In contrast, TNF-α KO BM had significantly less microvessel density, as shown by the expression and quantification of basement membrane markers laminin and collagen IV (Figure 7B, p<0.05). The BM of 3xirradiated mice also presented increased MMP-2 and MMP-9 (Figure 8A), VEGF and NFkB p65 expression (Figure 8B), while in control WT or TNF-α KO mice these parameters remain unchanged. In addition, *ex-vivo* TNF-α treatment of isolated BM cells induced VEGF production (data not shown). Taken together, these data suggest that in 3xirradiated mice sustained BM TNF-α levels induce MMP activity, VEGF production/release and NFkB expression, thereby promoting disease progression.

## Discussion

In the present report we exploited the possibility that irradiation, known to induce BM malignant transformation, namely secondary acute myeloid leukemias [20] and MDS [21], might act as an inducer of TNF-α in the BM microenvironment, and studied the consequences of such an increase in the incidence of BM cell apoptosis and subsequent onset of BM dysfunction.

First, we observed that the effects of whole body “short term irradiation”, resulting in BM cell turnover and subsequent recovery, correlated with the levels of BM TNF-α. Moreover, *in vitro* exposure to supernatants from irradiated BM stroma, treated or untreated with a neutralizing antibody to TNF-α, demonstrated the TNF-α released in response to irradiation is partially responsible for BM cell apoptosis induction. Other studies have highlighted the role of TNF-α signalling in hematopoietic progenitor turnover *in vitro* [22]–[24]. Accordingly, we demonstrate *in vivo* thatWT mice treated with anti-TNF-α Ab as well TNF-α deficient mice were more resistant to irradiation-induced BM cell turnover than non-treated WT mice. Irradiation-induced production of TNF-α have also been reported in other systems like lung [25], [26], brain [27] and epidermis [28], [29] and related with malignant transformation.

We developed a model of BM dysfunction induced by prolonged (long-term) irradiation exposure, to study the importance of BM TNF-α in this setting. Our aim was to establish a reproducible and clinically relevant model of BM dysfunction that resembled secondary (that is, resulting from therapy) MDS. It has been known for several years, that dogs chronically exposed to low daily doses of whole-body γ-radiation are prone to develop hematologic changes consistent with a myeloproliferative disorder [30], [31]. In our 3-cycle irradiation model (3x irradiation), 40–50% of WT mice presented low WBC, anemia and thrombocytopenia, and elevated MCH-Hemoglobin per RBC (indicative of macrocytic anemia). These clinical features, together with the incidence of cytogenetic abnormalities (which in our *in vivo* model involved the loss of microsatelite markers in chromosome 2) are strong indications of an MDS-like phenotype [19]. TNF-α KO mice subjected to the same irradiation protocol did not develop BM dysfunction; cell apoptosis in TNF-α KO mice BM was significantly reduced, resulting in sustained hematopoietic precursor and mature cell levels, including MK. Besides the maintenance in MK/platelet levels, the circulating WBC and RBC in 3x irradiated TNF-α KO mice were also similar to control (non-irradiated) mice (data not shown). Taken together, these data strongly suggest that TNF-α KO mice are resistant to irradiation-induced BM dysfunction.

WT mice with sustained MDS-like symptoms beyond 5 months after the last irradiation had increased BM angiogenesis, as determined by quantification of laminin staining in BM sections and also by quantification of collagen IV and laminin in whole BM extracts (since laminin and collagen IV are components of the vessels basement membranes), suggestive of disease progression. The BM of 3x irradiated WT mice also showed increased MMP activity, increased VEGF and NFkB p65, in contrast to the BM of 3x irradiated TNF-α KO mice, where these parameters were unchanged. TNF-α is an important modulator of MMP activity, namely gelatinaseB/MMP9 [32], [33] which is crucial for extracellular matrix remodelling and has also been shown to play a pivotal role in BM recovery following myelosuppression [34]. We determined the levels of MMP released by irradiated BM stroma in the presence or absence of a TNF-α neutralizing antibody *in vitro*. Under these conditions, MMP-9 activity in culture supernatants was diminished in the presence of the TNF-α antibody (data not shown) suggesting TNF-α release from the irradiated stroma induces a rapid MMP release.

Therefore, besides inducing cell apoptosis, overproduction of TNF-α in the BM microenvironment following irradiation, may induce MMP-9 and possibly other undisclosed factors, contributing towards degradation of the endothelial basement membrane and release of pro-angiogenic factors such as VEGF, which in turn may promote angiogenesis. Moreover, our data also shows that TNF-α stimulates VEGF production and release from different BM cells, in particular MK. Concerning the involvement of NFkB, TNF-α has been shown to exert some of its effects via NF-kB [35], [36] The increase in NFkB p65 in 3x irradiated mice suggests this may be a molecular event involved in BM dysfunction and possibly progression to an MDS-like phenotype with increased likelihood of developing acute leukemia.

Our recent unpublished data suggested that MK are major producers of ECM molecules in the BM microenvironment (manuscript in preparation) namely fibronectin, suggesting their survival, observed in TNF-α KO mice, and the resistance of TNF-α KO mice to the effects of irradiation, may involve the production (maintenance) of such ECM molecules in the BM microenvironment. Lack of integrin α4 (which, among other functions is a receptor for fibronectin) has been shown to restrict stem-cell BM repopulating capacity and to limit BM stem cells self-renewal [37].

Two recent elegant studies demonstrated unequivocally that the interaction between hematopoietic stem cells (HSC) and the BM microenvironment is strictly regulated by Rb and retinoic acid receptor signalling [38], [39]. In both situations, the absence of such proteins resulted in the development of myeloproliferative diseases; interestingly, in the case of retinoid acid deficiency, transplanting total BM from TNF-α KO into retinoic acid receptor deficient mice protected these from myeloproliferative disease. The authors concluded that TNF-α is one of the mechanisms involved in BM malignant transformation, particularly in the case of myeloproliferative disease. The data shown in our present report demonstrates that TNF-α regulates BM cell turnover (apoptosis) induced by irradiation, conditioning the onset of BM dysfunction and secondary MDS-like phenotypes; moreover, our data also highlight a role for TNF-α in modelling the BM microenvironment, contributing towards the progression of secondary MDS.

Strategies to block TNF-α activity, seeking to neutralize its inflammatory role, have met some success in the treatment of epithelial cancers, and to a lesser extent also in subsets of patients with primary MDS, although with varying degrees of therapeutic benefit [15]–[17], [40]. The fact that TNF-α is a key regulator of BM cell apoptosis provided the rationale for the use of TNF-α blockers in primary MDS. Anti-cytokine therapy (amifostine or pentoxifylline and ciprofloxacin with or without dexamethasone) have been administered to primary MDS patients and the ones with high BM TNF-α levels have a better chance of responding to such therapy [41], [42]. Interestingly, in the reported trials patients with secondary MDS were excluded. The present report shows the apoptotic effects of TNF-α may be crucial for BM dysfunction and the onset of secondary MDS; moreover, since TNF-α (directly or indirectly) regulates ECM turnover and angiogenesis (as shown in our present study), it may also promote secondary MDS progression.

Taken together, we suggest that BM TNF-α is a critical factor in the onset and subsequent progression of irradiation-induced BM dysfunction with clinical features of secondary MDS (shown *in vitro* and *in vivo*), and as such, strategies designed to block the effects of TNF-α in the BM microenvironment may be an attractive option to treat patients with secondary MDS.

## Materials and Methods

### Mice

TNF-α knock-out (KO) and wild-type (WT) mice were obtained from the Jackson Laboratory, maintained on a C57BL/6 background. Animals were kept under specific pathogen-free conditions and handled in compliance with the Instituto Gulbenkian de Ciência or the IBMC-INEB Animal House guidelines and the European Convention.

### Bone Marrow Recovery Model

To determine the effects of short term irradiation in BM turnover and cytokine induction, 10 week-old WT mice were sub-lethally irradiated (300 cGy) and BM collected (from flushing one femur cavity with PBS) at different time-points after irradiation (6 h–72 h, 7 days). Cell suspension was incubated with red cell lysis buffer (RCLB) for 15 minutes, centrifuged at 1200 rpm for 5 minutes and the supernatant (mononucleated BM cells) collected for further analysis.

### *In Vivo* TNF-α Neutralization

The TNFα neutralizing antibody was delivered to 10 week-old WT males by intraperitoneal injections of 1 mg daily, beginning two days prior sub-lethal irradiation. Control animals received equivalent amounts of PBS. Following irradiation, BM cells were isolated at different time-points as described above and processed for flow cytometry.

### Apoptosis Determination

The percentage of apoptotic cells during BM recovery following irradiation was estimated by flow cytometry in TNF-α KO or WT (neutralized or not with anti-TNF-α antibody) mice. Total BM mononuclear cells were double-stained with Annexin V-PE (BD Biosciences) and FITC-conjugated antibodies against lineage markers, such as CD11b (Biolegend) and Sca1 (BD Biosciences) for hematopoietic mature (mostly myeloid) and precursor cells, respectively. The cells where analyzed on a FACSCalibur flow cytometer, using the “Cell Quest” software.

### BM Dysfunction/MDS Model

Ten week-old TNF-α KO and WT mice were irradiated sub-lethally monthly over a three month period (3x irradiation). Haematological parameters (white blood cells, red blood cells, platelet levels) from irradiated and control mice were determined 7 days after each irradiation and 3–6 months after the last irradiation. Peripheral blood was collected from the cheek pouch in EDTA-coated tubes (Multivette 600, Sarstedt, Nümbrecht, Germany) and analyzed with a Hemavet 950FS cell counter (Drew Scientific, Oxford, CT, USA). Apoptotic cells in frozen and paraffin-embedded BM sections, obtained from mice sacrificed at the same time-points defined above, were detected by transferase-mediated dUTP nick-end labeling (TUNEL) assay. We used the in Situ Cell Death Detection Kit, POD and DAB Substrate Kits (Roche), following the manufacturer's instructions. The percentage of apoptotic cells was determined by counting stained (TUNEL positive) nuclei in a total of six high power fields (200× magnification) per condition. These quantifications were done in triplicate (3 independent counts/BM section).

Finally, when exhibiting signs of disease (such as weight loss, reduced mobility and starry coating) animals were sacrificed by CO2 asphyxiation, their BM and other internal organs removed and processed for further analysis as described below. As a control, healthy non-irradiated mice were sacrificed at the end of the experimental period, approximately 6 months after the last irradiation. Isolated BM cells or BM sections were obtained and analysed as described below.

### Cell Cultures and Irradiation Assay

Total BM mononuclear cells from WT mice and murine stromal cell line S17 were cultured in complete RPMI medium, 10% fetal bovine serum (Gibco BRL), overnight (o.n.) at 37°C. For all experimental procedures, serum was removed from the cultures. To determine whether irradiation induced TNF-α from BM cells, and whether this induced TNF-α leads to BM cell apoptosis, these BM mononucleated cells were irradiated with 1200 cGy; some of them were previously incubated with anti-mouse TNF-α/TNFSF1A antibody (R&D Systems), at a concentration of 0.06 µg/mL. Twenty four hours after irradiation, supernatants were collected and added to new BM mononuclear cell cultures which were incubated or not with the anti-mouse TNF-α/TNFSF1A antibody and irradiated (1200 rad) or not (controls) on the following day. At the end of the experiment, the incidence of apoptosis in BM cells submitted to different conditions was determined by flow cytometry (see above).

### TNF-α and VEGF ELISA

TNF-α and VEGF protein levels were determined by enzyme-linked immunosorbent assay (ELISA). Briefly, supernatants from irradiated and non-irradiated BM cells (isolated for lineage surface markers CD41 (MK), CD11 (myeloid), Flk1 (endothelium) and sca1 (hematopoietic precursors) and from cultured stromal cells were collected, and loaded onto an ELISA kit (Calbiochem) following the manufacturer's protocol.

### Counterstaining and Immunohistochemistry

BM from TNF-α and WT mice were fixed with 2% paraformaldayde in 0,12 M phosphate buffer pH 7.2 o.n. and frozen on dry ice embedded in 0,12 M phosphate buffer pH 7.2, 15% sacarose, 7.5% gelatine.

Eight µm sections from frozen BM were stained for basement membranes with anti-laminin (Sigma), 1∶100, and anti-CD41-FITC for megakaryocytes (Chemicon) antibodies. Briefly, sections were incubated with primary antibodies diluted in PBS, 0.1% BSA, o.n., 4°C, incubated in secondary antibodies, anti-rabbit Alex Fluor 488 (Molecular Probes) diluted 1∶1000 in PBS, 0.1% BSA. At the end, slides were mounted on Vectashield mounting-medium with Dapi (Vector) and analyzed by fluorescence microscopy on an Axioplan Microscope (Zeiss).

### RQ-PCR

Total RNA was extracted from mononuclear BM cells and cDNA synthesized following conventional protocols. D2Mit447 probe was design for a microsatellite located in a highly instable region of chromosome 2, prone to be lost after irradiation. TNF-α and D2Mit447 microssatelite mRNA quantification was performed by real time polimerase chain reaction (RQ-PCR) using the SYBR Green Master Mix kit (Applied Biosystems). Mouse β-actin gene (endogenous housekeeping gene) was used as standard reference (normalizer). The relative expression of TNF-α and D2Mit447 was calculated by using the comparative threshold cycle (CT) method.

### Protein Expression

Total protein extracts from all BM were obtained by lysing the samples in cold RIPA buffer (50 mM Tris-HCl, 150 mM NaCl, 1 mM EDTA, 1% Triton X-100, and 0.1% SDS), in the presence of protease and phosphatase inhibitors. After 30 minutes on ice, lysates were centrifuged for 15 minutes at 4°C and 14000 rpm. Equal protein amounts from each sample were used for dot-blotting and western blotting assays.

For dot-blotting, primary antibodies were anti-human fibronectin (Sigma), 1∶500, anti-laminin (Sigma), 1∶250, anti-collagen IV, (Chemicon), 1∶100 and anti-β-actin (Sigma), 1∶250.

For Western blotting the following antibodies and respective dilutions were used: anti-VEGF (Santa Cruz), 1∶400, anti-NFkB p65 (Santa Cruz), 1∶1000, and anti-β-actin (Sigma), 1∶250.

For both assays, nitrocellulose membranes were blocked in PBS, 1% BSA, 0.1% Tween-20 or PBS, 5% milk, depending on the antibody manufacturer's protocol, for 1 hour at room temperature. Secondary antibodies were anti–rabbit IgG-HRP (Santa Cruz), anti–mouse IgG-HRP (Santa Cruz) and anti–goat IgG-HRP (Santa Cruz), all at 1∶6000. The electrochemiluminescence (ECL) detection system (Amersham Biosciences) was used to visualize the presence of specific proteins on the nitrocellulose blots. The blots obtained by dot-blotting were quantified with Image J program and represent relative expression levels of each protein.

### Gelatin Zymography

Presence or absence of latent or active species of MMP-2 and −9 were monitored by gelatin zymography. Briefly, equal volumes (40 µl) of supernatant obtained from cell cultures, both from bone marrow mononucleated and stromal cells were mixed with 5x sample buffer (0.5 M Tris-HCl, pH 6.8, 5% sodium dodecyl sulfate, 50% glycerol, and bromophenol blue) and loaded onto a 10% sodium dodecyl sulfate-polyacrylamide gel containing 0.12% gelatin (Sigma Chemical Co.). The gel was run under nonreducing conditions at constant voltage of 180 V for 5 hours. To remove SDS and allow the MMPs to renuture, the gel was incubated in 2.5% Triton X-100 at room temperature for 1 h in an orbital shaker, and then incubated in low salt collagenase buffer (50 mM Tris, pH 7.6, 5 mM CaCl2, 0.2 M NaCl and 0.02% Brij-35) at 37°C, overnight. To reveal the lysis zones, the gel was stained in stain/destain solution for 30 minutes, and washed in distilled water.

### Statistical Analysis

Results are expressed as mean ± standard deviation. Data were analyzed using the unpaired two-tailed student's t test or the one-way ANOVA Turkey test. P values of <0.05 were considered significant.

## Supporting Information

Figure S1 Irradiation induces the loss of microsatelite markers. Total BM cells were obtained from control and 3xirradiated mice. The results show the loss of microssatelite markers (D2Mit230) in irradiated mice BM cells, suggesting the irradiation protocol induces chromosomal abnormalities in irradiated mice bone marrow cells. *: p<0.01
(0.20 MB TIF)
