# Supplementary figures and images for: TNF-α Regulates the Effects of Irradiation in the Mouse Bone Marrow Microenvironment
Source: PLoS One. 2010 Feb 1;5(2):e8980. doi: 10.1371/journal.pone.0008980 (PMC2813873; doi:10.1371/journal.pone.0008980)

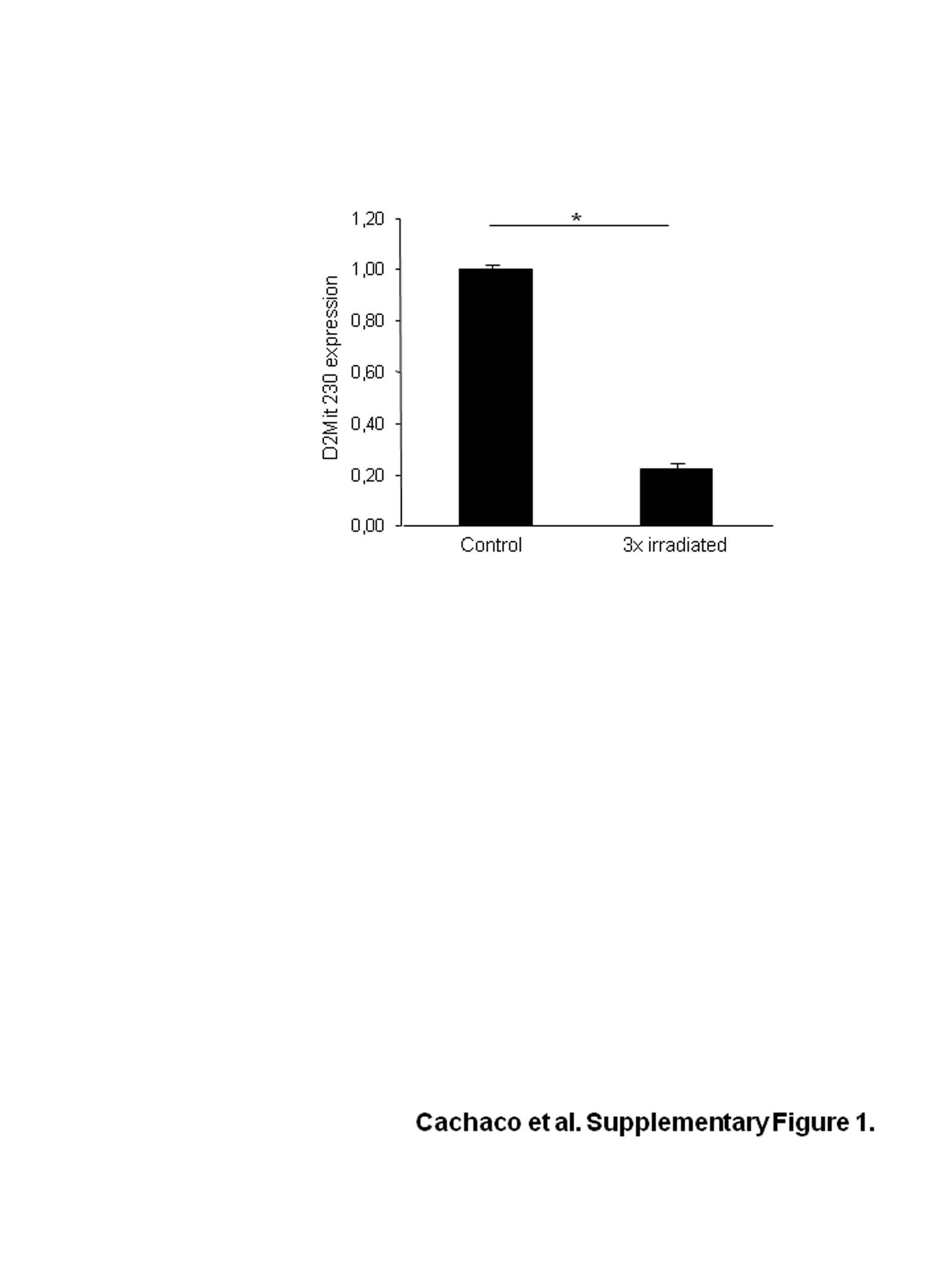

Supplement: Figure S1 — Irradiation induces the loss of microsatelite markers. Total BM cells were obtained from control and 3xirradiated mice. The results show the loss of microssatelite markers (D2Mit230) in irradiated mice BM cells, suggesting the irradiation protocol induces chromosomal abnormalities in irradiated mice bone marrow cells. *: p<0.01 (0.20 MB TIF) [file pone.0008980.s001.tif]
